# PCA, PC-CVA, and Random Forest of GCIB-SIMS Data for the Elucidation of Bacterial Envelope Differences in Antibiotic Resistance Research

**DOI:** 10.1021/acs.analchem.4c02093

**Published:** 2024-08-20

**Authors:** Alfred Fransson, Kelly Dimovska Nilsson, Alex Henderson, Anne Farewell, John S. Fletcher

**Affiliations:** † Department of Chemistry and Molecular Biology, University of Gothenburg, 405 30 Gothenburg, Sweden; ‡ Centre for Antibiotic Resistance Research (CARe), University of Gothenburg, 413 45 Gothenburg, Sweden; § Faculty of Science and Engineering, The University of Manchester, M13 9PL Manchester, United Kingdom

## Abstract

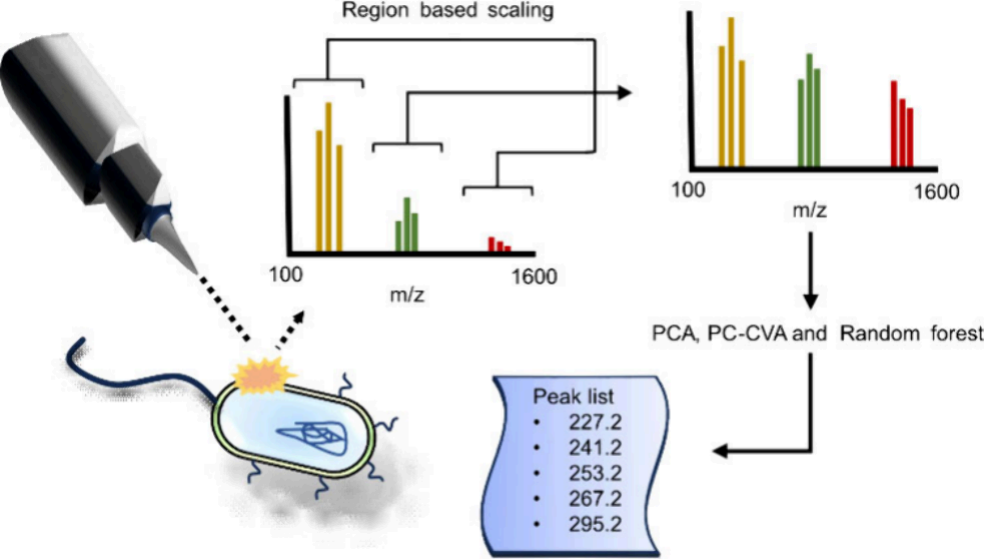

Antibiotic resistance can rapidly spread through bacterial populations via bacterial conjugation. The bacterial membrane has an important role in facilitating conjugation, thus investigating the effects on the bacterial membrane caused by conjugative plasmids, antibiotic resistance, and genes involved in conjugation is of interest. Analysis of bacterial membranes was conducted using gas cluster ion beam–secondary ion mass spectrometry (GCIB-SIMS). The complexity of the data means that data analysis is important for the identification of changes in the membrane composition. Preprocessing of data and several analytical methods for identification of changes in bacterial membranes have been investigated. GCIB-SIMS data from *Escherichia coli* samples were subjected to principal components analysis (PCA), principal components–canonical variate analysis (PC-CVA), and Random Forests (RF) data analysis with the aim of extracting the maximum biological information. The influence of increasing replicate data was assessed, and the effect of diminishing biological variation was studied. Optimized *m*/*z* region-specific scaling provided improved clustering, with an increase in biologically significant peaks contributing to the loadings. PC-CVA improved clustering, provided clearer loadings, and benefited from larger data sets collected over several months. RF required larger sample numbers and while showing overlap with the PC-CVA, produced additional peaks of interest. The combination of PC-CVA and RF allowed very subtle differences between bacterial strains and growth conditions to be elucidated for the first time. Specifically, comparative analysis of an *E. coli* strain with and without the F-plasmid revealed changes in cyclopropanation of fatty acids, where the addition of the F-plasmid led to a reduction in cyclopropanation.

## Introduction

The introduction of gas cluster ion beams (GCIBs) for secondary ion mass spectrometry (SIMS) analysis has been a disruptive technology, changing the expectation of the type of information delivered from a SIMS experiment. More intact molecular ions are detected, and the typical working mass range has been increased with ions regularly detected at *m*/*z* > 1000. Importantly, this is achieved while maintaining the surface sensitivity of SIMS and reducing subsurface damage accumulation, allowing intact molecules to be detected even after prolonged ion beam exposure. Despite this, the data is still complex, albeit richer than before, containing a mixture of intact species and fragment ions. Further, GCIB-SIMS is most commonly being applied for the analysis of biological samples such as cells and tissue samples that are also inherently extremely complex.^1−5^ The spatial resolution, surface sensitivity and depth profiling capabilities of SIMS has all been applied in different studies of bacteria and their biofilms.^6−11^ As such, the benefits of data reduction techniques, such as the use of multivariate analysis methods, have become even greater.

Principal components analysis (PCA) has been a go to technique for exploratory analysis of SIMS data for many years. As an unsupervised approach, PCA provides information about the main variation within the data set and can be used to identify patterns in chemical signatures for different samples, where a sample is a mass spectrum. PCA is sensitive to data pretreatment, including different scaling methods. Supervised multivariate analysis methods such as principal components discriminate function analysis (PC-DFA, also call principal components canonical variate analysis, PC–CVA) and partial least-squares–discriminant analysis (PLS-DA) have not been as widely applied to SIMS data although there are examples of supervised analysis, e.g., for classification of biological samples including bacteria, predominantly based on fragment ions.^12−18^ More recently machine learning approaches have been introduced for analysis of different data types including MS data.^19^ Of particular interest in the SIMS field has been the introduction of Random Forests^20^ as, unlike many machine learning/AI approaches, Random Forests can provide an output of the peaks used for classification that can be used for further investigation.^21,22^ Hence, as with PCA and PC-CVA, Random Forests provide chemical information about potentially important differences in the data set that can be investigated further, thus maintaining the strength of mass spectrometry as a discovery technique.

In this paper, we investigate the use of unsupervised (PCA) and supervised (PC-CVA) multivariate statistical approaches and supervised machine learning (Random Forests) to extract pertinent biological data from GCIB-SIMS data containing mixed molecular and fragment information. The data analysis approaches are applied and optimized for data acquired from bacterial samples with varying degrees of surface specific biological alterations. The data analysis is then challenged with respect to the number of replicate samples, degree of random variation, and the ability to extract very subtle changes in biology.

Many bacteria carry plasmids that are extrachromosomal elements which are not part of the cells’ core genome. Conjugative plasmids carry genes that encode structures and systems that allow the plasmid to be transferred from one cell to another via conjugation. Conjugative plasmids often carry antibiotic resistance genes that, when spread into a bacterial population, can mean that the population becomes resistant to certain antibiotics. All the strains used in this work have previously been used to investigate bacterial conjugation in *Escherichia coli* (*E. coli*).^23,24^ In the previous work, novel genes were found that negatively impact conjugation upon deletion. A subset of the identified genes was related to the membrane, this included *fabF*, which is involved in fatty acid elongation. There is an interest to understand the mechanisms behind genes governing conjugation and the role of the bacterial membrane in an effort to combat the spread of antibiotic resistance through inhibition of conjugation. However, the effect of the conjugative plasmids on the bacterial cells can be quite small and sometimes hard to detect. In this paper we investigate and optimize methods for analyzing GCIB-SIMS data first on data from a *fabF* deletion strain, impaired in fatty acid elongation and known to be deficient in conjugation. Further we apply these findings to reveal subtle changes in the bacterial envelope as a result from the presence or absence of the conjugative F-plasmid in the cells.

## Materials and Methods

The GCIB-SIMS data were extracted from SIMS images that had previously been interrogated using a combination of image analysis, manual peak comparison and univariate statistics.^25^ The data included an *E. coli* KEIO^26^ deletion *fabF* mutant strain with a deletion in the *fabF* gene, and three strains previously used as controls in previous work, also found in Table S1.^24^ The *fabF* mutant carries the conjugative extrachromosomal F-plasmid, which produces a transport system to allow for transfer of copies of itself from one cell to another. The F-plasmid also carries a tetracycline resistance gene. In this new work, the controls are instead called Conditions keeping the abbreviations C1–3 used in the previous work.^24^ All the strains in this work were cultured in LB and kanamycin. Condition 3 (C3) is the reference/control strain also known as HA14^23^ and grown in the same conditions as the *fabF* mutant strain. The control bacteria (C3), like the *fabF* mutant strain, carry an F-plasmid that confers tetracycline resistance. Condition 1 (C1) is the same strain as used in C3, carrying the F-plasmid but grown without tetracycline. Condition 2 (C2) is the same bacterial background as C3, but it lacks the F-plasmid and was thus grown in the absence of tetracycline. The data were acquired from three repeated experiments, each with four biological replicate bacterial droplets that had been washed with ammonium formate prior to being spotted on a silicon wafer and then analyzed in two areas for each droplet. This resulted in a total of 48 spectra across all experiments.

Analysis was performed on a J105 SIMS instrument (Ionoptika Ltd., U.K.) using a 40 keV (CO_2_)_6k_^+^ GCIB This cluster size was selected based on previous optimization experiments on Irganox1010.^27,28^ The ion dose was 1 × 10^11^ ions/cm^2^. Mass resolution (*m*/Δ*m*) was ca. 5000 at *m*/*z* 761. Spectral data were extracted from the SIMS images and imported into MATLAB (R2022b, MathWorks, Natick, Massachusetts). The automated process of peak picking and centroiding were performed using the ChiToolbox.^29^ Improving the mass resolution would allow more peaks (variables) to be resolved for the subsequent data manipulation and analysis that was performed within MATLAB using the ChiToolbox at a detriment to the memory requirements and processing time. All MATLAB code and links to the toolbox can be found in the (SI, methods 1). Briefly, the spectra were separated into three regions. The regions were selected visually based on the intensity pattern of the spectrum. Each region was then picked for peak picked. Then either (1) the peak picked regions were recombined into a single spectrum and then normalized to the sum of selected peaks in the combined spectrum or (2) each region was normalized separately before recombination into one spectrum. Post normalization the recombined spectra were then square rooted to reduce the dynamic range (SI, methods 2).

The PC-CVA analysis was performed by using the first 10 principal components (PCs) to generate a number of canonical variates (CV). The number of CVs produced equals the number of groups -1 (degrees of freedom). For the RF an 80/20% training/test split was used, where the model used 80% of the data to construct the model that was then tested on the last 20%. Additionally, the RF model was constructed using 1000 decision trees. The number of trees used depends on the size of the data set with larger data sets containing more samples benefiting from more decision trees but also leading to increased computing times.^30^

To assess the different data analysis approaches, we focused on the *fabF* mutant as the biological changes associated with this mutation are the most discernible with SIMS and thus provide a useful reference for method development and validation. Additional analysis to elucidate the effect of growth with tetracycline and the presence of the F-plasmid on the bacterial cell surface with a low expected detectable variation was also performed.

## Results and Discussion

### Region Scaling of Spectral Data Maximizes High Mass Contributions to MVA

The implementation of GCIBs has resulted in SIMS being a reliable way to perform molecular profiling of complex biological samples, with minimal subsurface damage allowing for analysis with high primary ion beam dose. The use of GCIBs further results in lower fragmentation and allows for improved detection of intact lipids and cardiolipins.^28,31^ While analysis with a GCIB is softer than conventional atomic or small cluster ion beams, it is still not a true “soft ionization” approach and a large degree of fragmentation can still be present in that mass spectrum particularly at low *m*/*z*.

The large amount of data generated during GCIB-SIMS analysis makes manual analysis of the data time-consuming and sometimes confusing. Multivariate analysis (MVA) methods can be utilized to help with visualization and interpretation of the data. MVA methods such as PCA can be used to extract information about overall variation within a data set. PCA reduces the dimensionality of big data sets and combines multiple variables (individual ion intensities) into new variables (principal components, PCs). Each PC has a corresponding loading that describes the contribution of the original variables to that PC.

**Figure 1:**
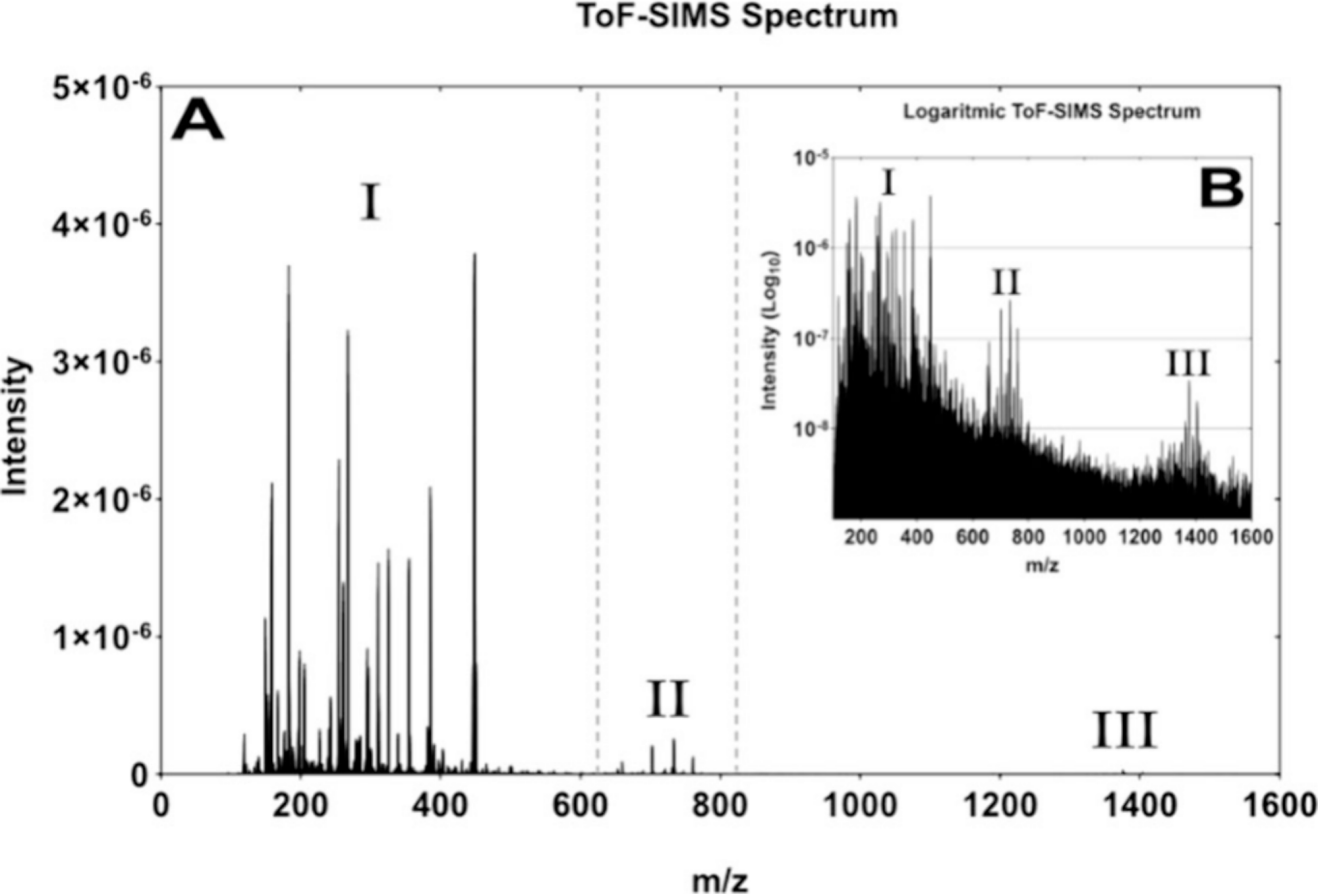
Typical GCIB-SIMS mass spectrum acquired from *E. coli* (A), while (B) is the same data shown on a log 10 scale. Three different regions of the spectra can be seen denoted by I, II, and III and separated by the dotted lines. These three regions are primarily fatty acids, lipids, and cardiolipins, respectively. A common trend can be seen in (A) and (B), where a 10-fold decrease in maximal intensities can be seen between I, II, and III in both images. This highlights the risk of meaningful information being overshadowed by the higher intensity regions.

A GCIB-SIMS mass spectrum recorded from the surface of an *E. coli* sample is shown in Figure 1. The acquired spectral data showed three mass regions in the *m*/*z* 100–1500 range with a 10-fold incremental decrease in intensities creating a stair-like pattern in the spectra (Figure 1). Region I contains low mass ions, many of which are expected to be fragments of larger ions, e.g., RCOO^–^ ions (typically in the *m*/*z* 150–400 range) from fatty acid groups that have separated from intact lipids. Region II contains intact phospholipid signals, while Region III contains signals from larger lipids, such as cardiolipins. This intensity pattern of these three regions is not only seen in *E. coli* but also in GCIB-SIMS data from mammalian cells and tissue samples, indicating that a similar approach can be used for various biological samples.^32^ Large general variations in intensity can be a problem when using analysis methods such as PCA where the intensity of the peaks is used to determine the variation between samples. High intensity low mass peaks can exhibit high variance, sometimes due to noise, and this can overshadow the variance from lower intensity, higher mass peaks. This can, in turn, result in important biological information being missed.

There have been several methods reported for scaling and reducing the overall dynamic range of SIMS data sets: for example, logarithmic scaling of the ion intensities or taking the square root of the intensities of the data.^33,34^ Scaling to account for Poisson counting and deadtime has been also demonstrated but may not be applicable to the analogue ion counting system used in this study.^35^

Additionally, to address the dominance of low-mass ions, mass (*m*/*z*) scaling has been used to enhance the signals from higher- mass ions. While this may be a useful general approach, it does not address the specific stepwise signal level changes observed from biological samples. In this work, we investigate the benefits of *m*/*z* region-based scaling of data and the effect on several data analysis methods: PCA, PC-CVA, and Random Forest.

### Comparison of PCA when All the Data Is Scaled Together or if the Data Is Region Scaled

While MVA methods provide a means for spectral classification, in a research setting where exploratory biological studies are common, MVA is often used as a starting point for prioritizing detailed annotation of the mass spectrum. As an exploratory (unsupervised) method, MVA can be used to identify regions of the spectrum that may be of interest to the analyst. In this work, a stepwise scaling approach is tested in terms of producing the most readily interpretable loadings containing the most useful biological information. It is difficult to define which loading gives the most useful biological information with a new set of data, so a data set was selected that has been previously subjected to detailed manual analysis.^24^ To assess different analysis methods, we chose to use a set of data that could be compared in a variety of ways. First, and most simply, we compared data from an *E. coli* mutant (*fabF*) which is altered in fatty acid synthesis, with the C3 control strain grown on the same day in identical conditions (see Materials and Methods).

The deletion of *fabF* limits the generation of FA(18:1) fatty acids during logarithmic growth which effectively leads to a reduction in the abundance of cyclopropanated FA(cp19:0) fatty acids once stationary phase is reached.^36^ These bacteria were analyzed in stationary phase, and therefore changes in the relative abundance of (cp19:0) fatty acid fragments and (cp19:0) containing intact lipid signals should be a significant biological difference between the bacterial samples.

**Figure 2:**
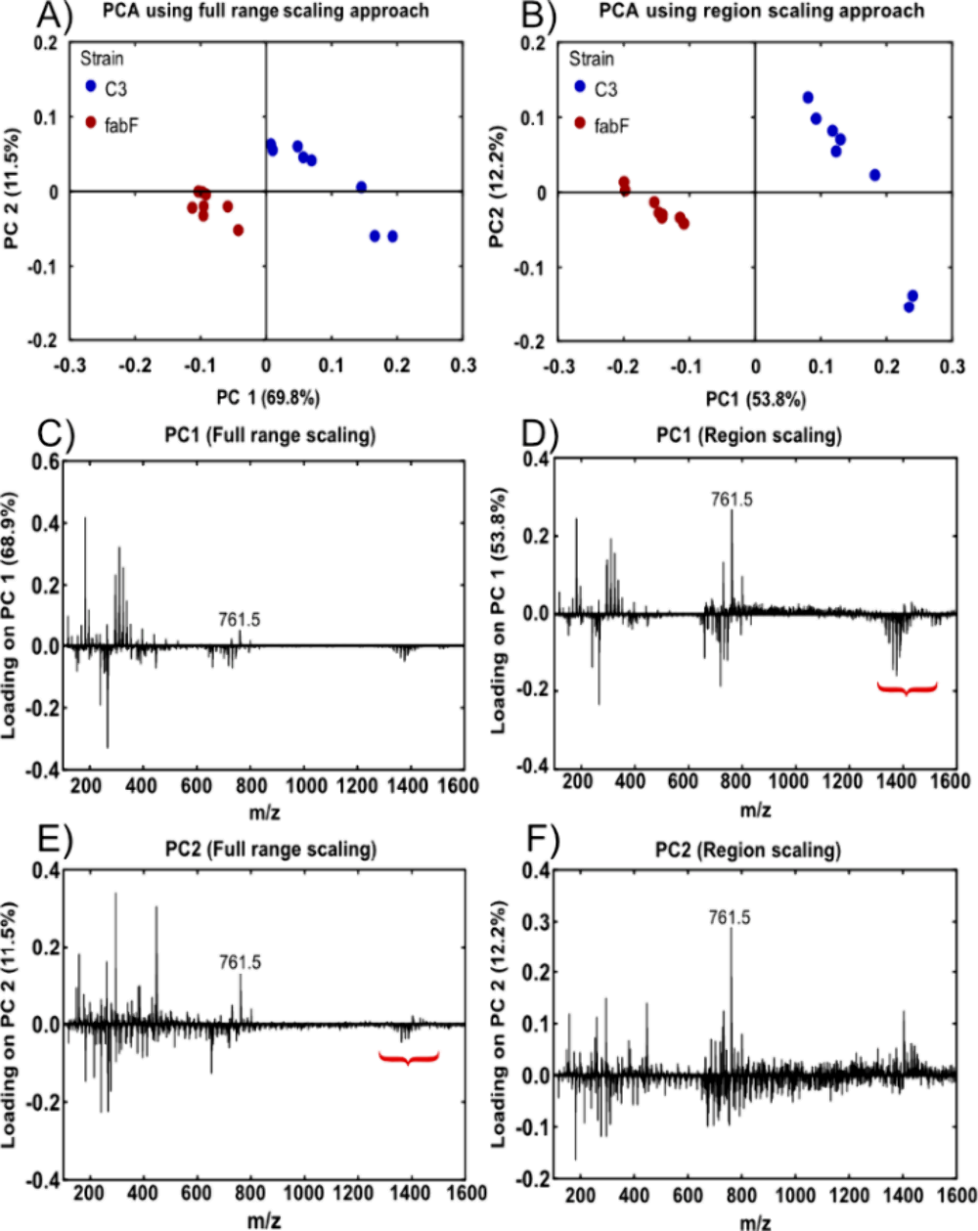
(A) PCA score plot of PC1 and PC2 of the data set containing data from one experiment comparing the C3 control and the *fabF* strain. (C) and (E) are the plots of the loadings of PC1 and PC2, respectively. (B) shows the PCA result from the same data set but instead using a region scaling approach where the spectrum is divided into three regions based on intensities and then each region normalized independently before being combined back into one full spectrum. The corresponding loadings of PC1 and PC2 for (B) can be seen in (D) and (F), respectively. Normalization of spectral regions separately can enhance features and decrease overshadowing by high intensity variables in PCA. The red bracket in (D) and (E) highlights a spectral feature of a split in the cardiolipin signals that was concentrated into PC1 when using the region scaling approach.

In this work, a new approach was tested using region scaling of data before multivariate analysis. The spectrum was first divided into *m*/*z* regions based on the differences in maximal intensities: Low mass (*m*/*z* 100–660), Mid mass (*m*/*z* 660–820), and High mass (*m*/*z* 820–1600) regions capturing the three general intensity steps in the mass spectrum, as shown in Figure 1. Each of the three subregions were peak picked separately. The picked peaks from each subregion were then normalized to the sum of selected peaks within the *m*/*z* subregion (sum normalized) before the three regions were recombined into one spectrum. This was done to balance regions in the total spectrum with the purpose of giving more weight to low intensity peaks at higher mass, which can exhibit a high variation in the analysis. As a final preprocessing step, the spectra were square rooted to reduce the nonmass dependent dynamic range of the data. PCA was performed on the region scaled data, and loadings examined. PCA was also done on the same data set, but normalization was instead applied to the full range spectrum and not the subregions as above. Thus, the effect of region scaling on the PCA loadings versus scaling the entire spectrum was compared. Figure 2 contains scores and loading plots for the two PCA results. Score plots using PCs 1 and 2 are shown in panels A and B for the full range and region scaled data, respectively. Separation of the two bacterial strains could generally be achieved on PC1 alone but an increase in the separation between the mutant strain and the control on PC1 was observed in the region scaled analysis. In addition, when region scaling was included, the contribution of higher *m*/*z* peaks such as the *m*/*z* 761.5 peak in PC1 (Figure 2D vs 2C) increased. This is important because this peak corresponds to PG(16:0)/(cp19:0) known to be absent in the *fabF* mutant.^37,38^

While the spectra are separated in the PC1 direction, improved clustering is observed when considering the PC2 contribution in both cases. One explanation for the improvement in separation between the data sets on PC1 is the appearance of spectral features present in the loading plot of PC2 for the full range data in the PC1 loadings of the region scaled data. For example, there was a split in the loading direction of different cardiolipins in the region scaled PC1 loadings (Figure 2D) that can be seen in the PC2 loadings (Figure 2E) of the full range data. Further, variation explained by high masses between *m*/*z* 600–1600 in the PC loadings and biologically significant spectral features, previously seen in PC2, was instead captured in PC1 when using region scaling.

By normalizing regions of a mass spectrum with large variation in intensity, we saw increased separation between the *fabF* mutant and the control strain along PC1 and there was less overshadowing of the low intensity peaks at higher mass by the high intensity peaks at low mass in the PC loadings. Based on our results, it was decided that region scaling as a first step was to be used for subsequent analytical approaches.

### PC Canonical Variates Analysis (PC-CVA) May Be Better When Class Information on Samples Is Known

PCA is a commonly used multivariate analysis method that uses unlabeled data to explore the relationship of the different data points without any applied bias and highlights the biggest variation within the data set. However, when looking for biological differences between two sample sets, a retrospective bias is applied in examining PCA results, possibly subconsciously, as one labels/color-codes the different sample groups in the score plots and visually looks for patterns (or specifically the pattern the analyst expects/hopes to see!). Supervised multivariate methods that apply *a priori* knowledge of sample classes provide a targeted approach to identifying specific differences between data sets. Supervised approaches have been used much less in SIMS data analysis than the unsupervised method, PCA, which is often employed for sample classification, such as in bacterial species determination.^12,39,40^

Hence, even though PCA is a commonly used way of analyzing and presenting differences between different samples instead one can use the supervised method Principal Components Canonical Variate Analysis (PC-CVA), which utilizes the PCs from the PCA (a dimensionality reduction step required prior to CVA, as CVA requires many more samples than variables) to identify latent variables called canonical variates (CV).^41^ The PC-CVA uses the known relationship or group membership of each sample based on labels to fit the variables into new CVs where the intragroup variation is minimized and the between groups variation maximized. Typically, in PCA, multiple PCs are used to visualize the difference between all samples in the data set, making identification of variables explaining said difference harder as one needs to analyze the loadings of multiple PCs. The PC-CVA uses the PCs to create a number of CVs equal to *n* – 1, where *n* is the number of samples/groups. This reduces the dimensionality of the data into fewer components, reducing the risk that spectral features spread over multiple PCs are missed during variable identification. When using PC-CVA, one can choose how many PCs to use. We empirically chose to use 10 PCs, but one can choose the numbers of PCs based on percentage variation captured instead.

**Figure 3:**
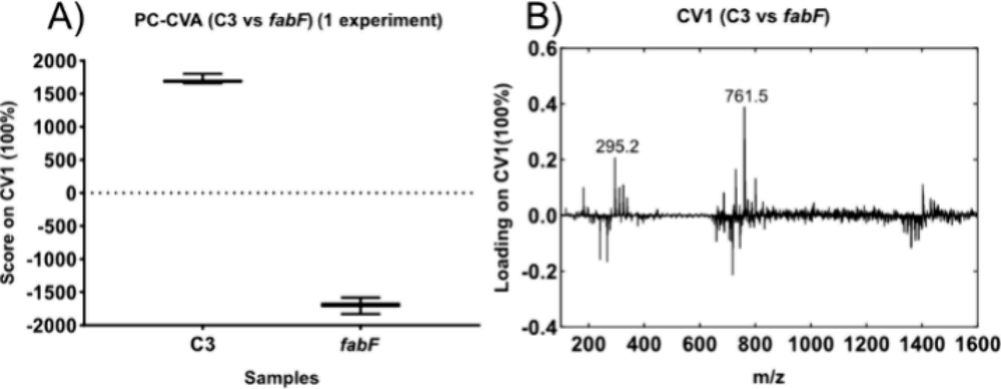
Result of a PC-CVA performed on the region scaled data from one experimental replicate of the *fabF* mutant and the C3 control. (A) Box plot of the PC-CVA score. The PC-CVA works well in separating the *fabF* mutant from C3 and the loadings of CV1 in (B) show a high weighting for peaks related to differences in lipids with an 18-carbon acyl chain (*m*/*z* 295.2 and 761.5), which are synthesized by FabF. In the PC-CVA of the region scaling here intact lipids at higher mass contribute more to the loadings than when PC-CVA was performed on the data, where the full spectra were normalized as one (Figure S1).

When comparing the region scaled PCA (Figure 2B) and PC-CVA (Figure 3A), it was seen, perhaps unsurprisingly based on the PCA result, that the PC-CVA was good at separating the mutant vs the control with the canonical variate 1 (CV1) using the same data set as used for the PCA. The loading from CV1 strongly resembles the loading from PC1 and this indicates that the biological variation within the group is low compared to the between group variation. In addition, CV1 incorporates features from multiple PCs (Figure 2D,F vs 3B). The PC-CVA assigns more contribution of the difference to the expected *fabF* related lipid peaks, e.g., FA(cp19:0) and PG(16:0)/(cp19:0) at *m*/*z* 295.2 and 761.5, respectively, between the samples.

In short, PCA can separate the control and *fabF* mutant strain. PCA explains the total variation within the data set and the separation can be seen with the first two PCs. The PC-CVA improves upon the PCA and can reduce the dimensions of several PCs to one CV leading to easier interpretation of the data and capturing expected biological changes for the *fabF* mutant, which was known to have large variations in the lipid composition within the cell envelope. As the CVA uses the PCs as a starting point then any improvements in the PCA result should continue to benefit the PC-CVA result. Specifically intact molecules are of an interest to us as fragmentation of larger molecules could yield the same fragments which can complicate the interpretation of the result. In the comparison of the region scaled data (Figure 3) versus the full range scaled data, as shown in the Supporting Information (Figure S1), the loadings of CV1 had a higher degree of variation contributed by the high mass, low intensity, ions using the region scaled approach.

PCA and PC-CVA worked well to explain the differences between the *fabF* mutant and the control, and the loadings looked similar to marginally better results in the PC-CVA. To reduce the variables in the initial MVA comparison, it was performed on a data set consisting of data collected on the same day. However, typically experiments should be done in replicates to reduce the chance of batch effects where environmental effects can lead to noise in the data. Hence, we decided to expand the data set with data collected from three independent experiments acquired over several months.

### PC-CVA Is More Robust than PCA When Including Replicate Experimental Data Acquired on Different Days

**Figure 4:**
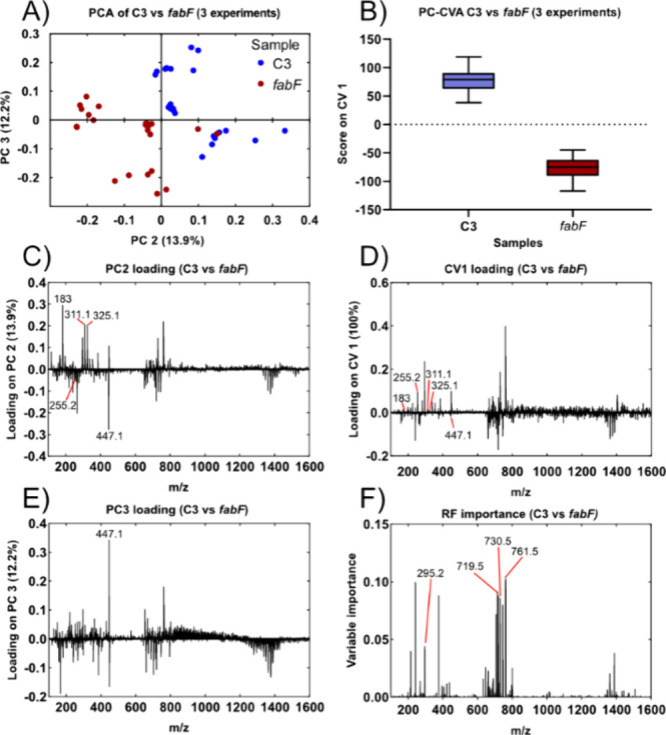
Comparison of PCA and PC–CVA performed on combined data from three replicate experiments. (A) PC score plot using PC2 and PC3. While there is a separation of the blue (C3) and red (*fabF*) strains there is still a clear grouping of the three experimental days. The corresponding loadings for PC2 and PC3 are shown in (C) and (E). (B) Box plot of the PC-CVA scores run on the same data set. In (D), the loadings of CV1, there are several peaks missing that can be found in the fatty acid mass range (*m*/*z* 200–350) of the PCA loadings and have been manually assigned as contaminants and likely responsible for some of the day-to-day variation seen in the PCA plot. (F) Shows the variable importance plot from a Random Forests analysis performed on the same data set showing some peaks detected in both the PC-CVA and the RF known to be affected in a *fabF* mutant.

Adding more data can improve the trustworthiness of the interpretation but can also introduce additional variation that can confound the MVA. To test the impact of additional data on PCA and PC-CVA, biological and technical replicates from two additional replicate experiments performed on different days months apart were included. The resulting score plot from the region scaled PCA using the data from all three experiments is shown in Figure 4A. Unlike the single experiment data set there is no longer separation on a single PC, and even with two PCs the separation is not perfect. Further, in the new case it is PC2 and PC3 (Figure 4A) that are now capturing the biological differences and not PC1 (Figure S2) as the day-to-day variation exceeded the biological variation. Day-specific subgrouping of the data is also evident even on these selected “biologically relevant” PCs.

When comparing the PC-CVA results from the data set containing data from three repeat experiments (Figure 4B) against a data set with only data from one experiment (Figure 3B), the relative difference in CVA score decreased in the score plot for CV1. The results of the PC-CVA indicate the presence of day-to-day variation, but the inclusion of two additional replicate experiments from different days allowed an averaging out of some of this variation, reducing the relative differences between samples. Adding multiple experimental replicates improved PC-CVA by reducing the batch effect to create an analysis more reflective of the true overall biological population variation. Some peaks in the single experimental CV1 loading were absent in the three-experiment CV1 loading. In the CV1 loadings from the analysis of multiple experiments (Figure 4D) some of the peaks present in the PCA loadings (*m*/*z* 183, *m*/*z* 311.1, and *m*/*z* 325.1; Figure 4C) are absent or greatly attenuated. These peaks have been determined to be surfactant contamination peaks, and inspection of the ion image data showed these peaks to be mainly localized to certain pixels in the image. Additionally, the *m*/*z* 447.1 peak observed in both the PC2 (Figure 4C) and PC3 (Figure 4E) loading was substantially reduced in the CV1 loading indicating a lack of biological importance. It was further observed that some masses would be weighted more in the PC-CVA three-experiment comparison, such as *m*/*z* 255.2, the RCOO^–^ ion of FA(16:0), which was not distinguishable when only looking at the PC-CVA of the single-experiment comparison but emerged when looking at the results of the three-experiment comparison. Manual inspection of *m*/*z* 255.2 in the ion images revealed changes in intensity between the mutant and the control despite this species not being explicitly affected by the mutation of the bacteria.

Overall, the inclusion of additional data acquired on different days in the PC-CVA retained most of the peaks from the analysis of the data from 1 day. In contrast, the PCA results using the three-experiment replicate data suffered from day-to-day variation and the inclusion of day dependent contaminants, which skewed the loadings of the PCs. This suggests that PC-CVA is more suitable than PCA for more complex data sets where additional replicates allow the analysis to average out some of the day-to-day variations and more strongly bring forth the biological variation explained, in this case, by the deletion of the *fabF* gene. The PC-CVA can handle more complex data sets that include more day-to-day variations better than PCA and produce loadings that highlight the main biochemical differences between two different groups.

### Random Forest as an Alternative Approach to PCA and PC-CVA

The increasing capabilities of machine learning have paved the way for new data-mining methods to help with increasingly complex data sets in the field of mass spectrometry.^19^ Random Forest (RF) is a supervised machine learning algorithm that uses knowledge about class membership to create a model for the classification of samples into different predetermined groups. Random forests are an ensemble method that uses an array of weak classifiers in the form of decision trees. During the construction of the model, many decisions trees are made; each tree is created using a subset of the variables of the data set. Each tree creates a classification path before being tested on unseen data. There is then a majority vote among all the trees, which results in the overall model’s answer to the outcome. The use of weak classifiers to build a good classifier, in this type of ensemble model, is called “bagging” for bootstrap aggregation.^20^ Based on the RF model, each variable is assigned an importance. The variable importance is a measurement of how often variables are used for classification and their significance in the prediction of the model. In addition, recently the importance has also been used as a way to determine the most important variables to explain sample differentiation.^22,42,43^ Hence, the importance values for each peak can be treated as loadings that explain which peaks are most important for classification, and so in this work the importances are used in an investigative approach for discerning differences, for example, between the *fabF* mutant strain and the control strain.

One noteworthy difference between the importance values and the loadings associated with PCA/PC-CVA is that the importance values do not provide an indication of whether the peak is relatively increased or decreased in the different data sets. Further, as the RF incorporates random elements each time it runs, the model will differ. The classification could be correct but be based on different peaks and thus produce different importance values. To achieve consistent results and to capture as many biological differences as possible, the RF was run multiple times on the same data set, and the importance was summed for each variable across all runs. During the optimization of the RF, we tested using 1, 10, and 100 consecutive runs. With 10 runs the resulting summed importance per variable became more consistent, and 100 runs did not show a noticeable improvement compared to using only 10. The optimum may be different for different data sets.

The RF approach was tested on bacterial data sets. The RF results from 10 runs could classify the different samples with a success rate of 100% even when only using data from 1 day. However, batch effects can occur when there are a low number of samples or replicates, which can cause nonbiological factors to play a bigger part of the outcome. It was observed that the RF importance plots were noisy and hard to interpret when looking only at data from one experiment. Further, because the RF uses part of the data in the data set as a training set to train the model, a low number of samples also means there is less information for the model to train on. The inclusion of additional experimental replicates from different days reduced the batch effect and allowed the RF to better train its model and reveal the biologically important peaks.

The RF results based on the data set of the three replicate experiments had similarities to the results of the PC–CVA with 26 out of the top 40 largest peaks being the same between the methods (Table S2). This includes the *m*/*z* 295.2, *m*/*z* 719.5, *m*/*z* 730.5, and *m*/*z* 761.5 (highlighted in Figure 4F), which were identified as important for differentiating between the *fabF* and control strain in previous work.^24^ When comparing the top hits for the PC-CVA the top 20 highest positive loading peaks and the 20 most negative loading peaks were used as the CV1 loading is multidirectional and thus has peaks going in both directions. There were some peaks that differed between the PC-CVA and RF but often peaks that only would appear in either the PC-CVA or RF analysis were peaks with relatively low intensities, making it harder to determine potential biological importance. It is worth noting that both the PC-CVA and RF include isotope peaks in their analysis and variables with the highest scores generally had multiple isotopes of the same molecule, and thus validation of generated peaks is recommended to determine which peak is the molecular ion peak. Overall, we concluded that both PC-CVA and RF worked well for the *fabF* versus C3 three-experiment comparison, used as our testing set.

The *fabF* mutant was known from previous work in our laboratory to have large specific decreases in lipids containing 18 carbon fatty acids^36^ and it was expected that most analysis methods would work reasonably well although we did see some improvements especially in the PC-CVA analysis.

### Small Variations in Biology Can Be Detectable Using GCIB-SIMS

To challenge the data analysis methods, a data set containing data acquired in the same way as in the *fabF* experiment was analyzed using PC-CVA and RF. This data were acquired from three conditions: the control used in the previous experiment Condition 3 (C3), which carry the F-plasmid (the F-plasmid carries tetracycline resistance) were grown in medium containing the antibiotic tetracycline, Condition 1 (C1) which is the same *E. coli* strain, but it was grown in the absence of tetracycline, and Condition 2 (C2) which is the same strain only lacking the F-plasmid carried by the original control strain thus, grown in the absence of tetracycline. These three samples/conditions are expected to be very similar biologically. However, it is known that there is a fitness cost in growth rate between the absence (C1) and presence (C3) of tetracycline in *E. coli* strains that carry resistance to tetracycline.^44^ This led us to suspect that there could be some minor differences in the membrane composition between the two conditions. In the case of having an F-plasmid (C1) or not (C2), without any environmental changes it is still suspected that there is a biological difference as the F-plasmid encodes for membrane embedded components of the bacterial conjugational machinery.^45^ We were interested in determining whether PC-CVA or RF could distinguish the different groups despite expected low variations and give interpretable loadings containing peaks showing a biological difference.

**Figure 5:**
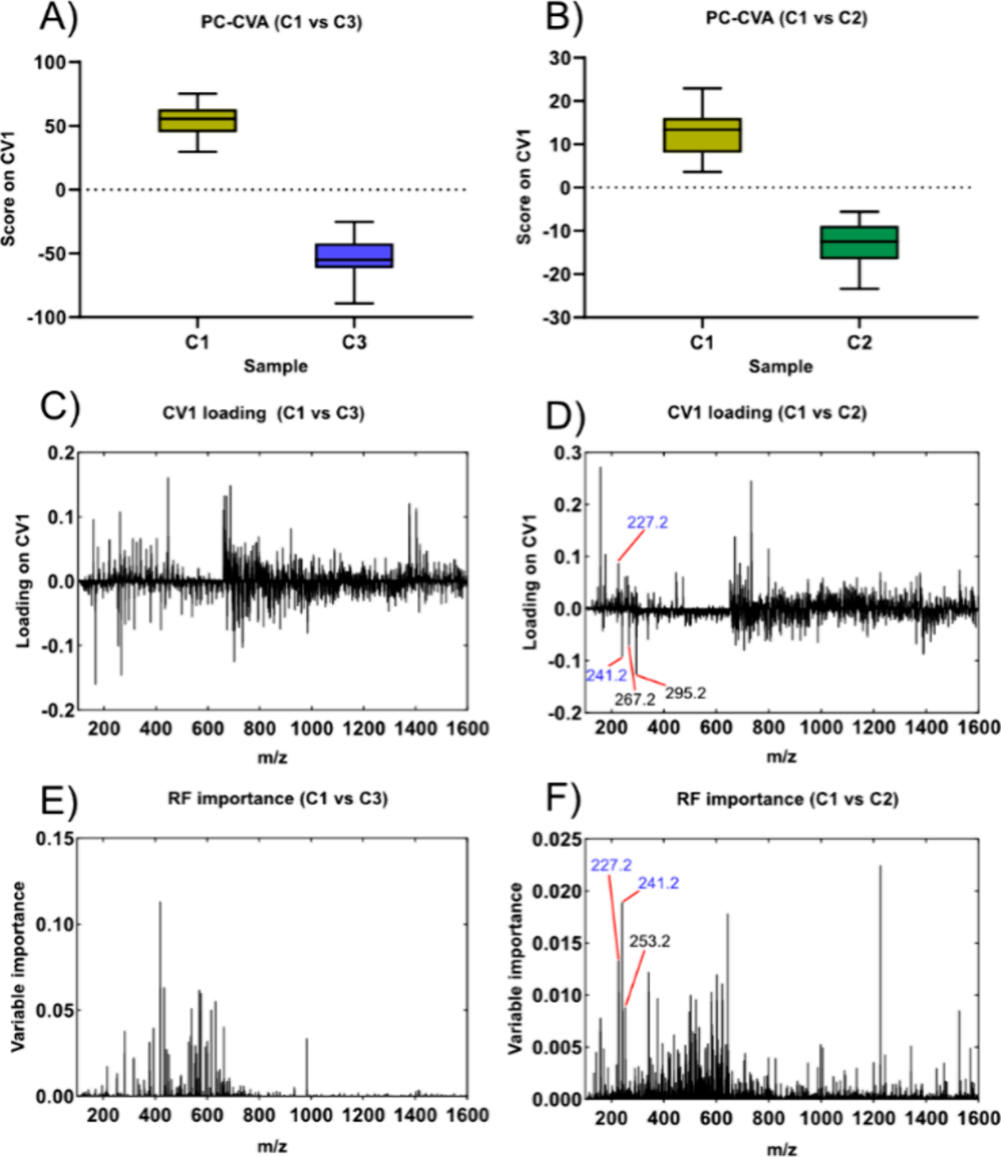
PC-CVA and RF analysis of the C1 and C3 conditions. (A) PC-CVA CV1 score as a box plot in the comparison of C1, which was grown without tetracycline, and C3, which was grown with tetracycline. (C) The loading of CV1 in C1 vs C3. (E) The resulting summed importance from 10 runs of RF. (B) The PC-CVA CV1 score as a box plot for the C1 vs C2 comparison, showing the lowest separation along CV1 in this work. (D) The corresponding loading of CV1, while (F) is the summed importance score from 10 runs of RF. The overlap in the top 40 peaks was only 10% (4 peaks; Table S4), but the overlap included several fatty acids and is based on the loading of the PC-CVA. It was observed that C1 had lower levels of the cyclopropanated fatty acids FA(cp15), FA(cp17), and FA(cp19) at *m*/*z* 241.2, *m*/*z* 267.2, and *m*/*z* 295.2, respectively.

First, the data comparing the tetracycline resistant wild-type strain grown in the absence or presence of tetracycline were analyzed (C1 vs C3, Figure 5A). One of the first things that could be seen was that the RF made some group classification errors but still maintained a percentage of correctly classified of around 95%. This is most likely a result of the differences between the samples being minor in comparison to the effect the deletion of *fabF* had. In the PC–CVA analysis, there was less separation between the C1 and C3 strains compared to *fabF* and C3 further indicating that C1 is more like C3 than *fabF* is to C3.

However, the loadings for the PC-CVA of C1 versus C3 (Figure 5C, 5D) are not as clear as compared to the PC–CVA with *fabF*. In the PC-CVA of the *fabF* mutant the loadings are less noisy and most weighting is attributed to a small number of peaks, this is likely because the *fabF* deletion affects FA(16:1) → FA(18:1) conversion which is a direct change in chemistry that is readily accessed by SIMS analysis. In the C1 vs C3 scenario there is a difference in the growth environment and the induced expression of the tetracycline resistance gene *tetA* has been known to have an impact on fitness.^46^ It stands to reason that something affecting the overall fitness of a cell will have a more widespread effect on the cells in general. The increase in noisiness and fewer standout peaks dominating the CV1 loadings could give an indication that growth with tetracycline causes a broader effect on the cells with more systems or components of the cells being affected and thus giving less specific biological variation. The overlap between the PC-CVA and RF also differed more in the case of the C1 vs C3 analysis compared to the *fabF* mutant, with 6/40 (15%) (Table S3) of the top 40 peaks appearing in both methods. The most influential peaks are also different between the methods, and manual validation of both methods showed that the most influential peaks from both methods showed a difference in the single ion images in the raw data.

The PC-CVA is a better initial visualization tool of the sample variation than RF as the separation could be used as a rough measurement for sample likeness. The RF could classify the samples to a high degree, but one of the negatives with using the importance for peak selection is that the trees used by the model for classification are created in a random manner. Thus, if only a few peaks are needed for efficient classification, then it might not include the variability of all peaks in its final model. However, the RF is a good addition to the PC-CVA as it might give a different perspective on which variables are important for determining differences in the samples even though it might not give the same inclusion as the PC-CVA which is based on all the peaks in the data set.

As a final analysis, a data set was used with the data acquired from the C1 and C2 strains where the only difference was that the C2 strain does not contain the F-plasmid, while both strains are grown without tetracycline. This data set was hypothesized to have the smallest differences between the different strains and conditions investigated here. The ability of the RF to correctly classify the different strains was the lowest among all the comparisons with an average classification rate of 85% across 10 runs of RF. The PC-CVA score plot also showed the lowest separation along CV1 (Figure 5B) among all the comparisons tested in this work. However, there was still enough separation to distinguish between the different groups.

This was surprising as we had doubts that there would be enough difference between the C1 and C2 strains for GCIB-SIMS to be able to detect the difference. The F-plasmid does encode for several membrane-bound components, but the number of the plasmid encoded proteins were believed to be small in the context of a whole cell. The overlap in the top 40 peaks from the RF and the PC-CVA was only 10% (4 peaks), but they included two fatty acids FA(14:1) and FA(cp15:0) at *m*/*z* 227.2 and *m*/*z* 241.2, respectively.

From tables of the top 40 hits for the RF and the PC–CVA it was also seen that several additional fatty acids were detected in one or the other analyses. This included FA(cp17:0) (*m*/*z* 267.2) and FA(cp19:0) (*m*/*z* 295.2) seen in the CV1 loading (Figure 5D) and FA(16:1) (*m*/*z* 253.2) seen from the RF (Figure 5F). Inspection of the CV1 loading and manual evaluation of the raw data revealed a consistent decrease in cyclopropanated fatty acids in C1, which indicates a lower degree of cyclopropanation in the strain containing the F-plasmid than in the one without the plasmid. As an additional test a *t* test was applied on the five mentioned peaks. The peaks seen in both methods, *m*/*z* 227.2 and *m*/*z* 241.2 were significantly changed with a *p*-value < 0.05. The *m*/*z* 253.2 peak seen in the RF also showed significance, while the two extra peaks from the PC-CVA did not show significance (Figure S3). It is worth mentioning that significance for a single peak might not always be relevant, as the purpose of this work is the extraction of overall biological features to warrant further investigations.

Cyclopropanation is typically seen as a response to stress as seen in membrane related stress caused by changes in temperature.^47^ A decrease in cyclopropanation could provide additional evidence that the F-plasmid can activate extracytoplasmic stress responses, indicating either an anticipated stress of conjugation or an overall increased membrane stress caused by the presence of the F-plasmid. It is known that the *cfa* gene encoding for cyclopropane fatty acyl phospholipid synthase which is responsible for cyclopropanation of unsaturated fatty acids in *E. coli* is downregulated by the *cpxQ* sRNA. *cpxQ* is part of the 3′UTR of the *cpxP* gene and the *cpxP* promoter is the most strongly induced promoter in response to induction of the cpx envelope stress response.^48^ An existing relationship between the F-plasmid and the cpx stress response system has previously been shown and indicates that the expression of the genes on the F-plasmid can cause membrane related stress.^49,50^ This may mean that an induction of the *cpxP* promotor leads to increased levels of *cpxQ* which in turn represses expression of *cfa* leading to decreased levels of cyclopropanated fatty acids.

It can also be observed that in the comparison of C1 vs C2 for both the PC-CVA and the RF there were fewer peaks that are distinguishable in the loading/importance plots, respectively, compared to the C1 vs C3 comparison (Figure 5). However, much like in the case of the comparison of C1 vs C3 PC-CVA, and the RF the overlap in the top hits is much less than with the *fabF* mutant. As observed when comparing C1 and C2, with and without the F-plasmid, some peaks that were important for determining the difference in cyclopropanation were only seen in one or the other analysis and so there is a benefit to doing both PC-CVA and RF. However, it also means that interpretation of the loadings/importance becomes harder and requires more manual validation of the results.

## Conclusions

Based on our results, we see a benefit of using region scaling as a preprocessing step when dealing with peak-picked GCIB-SIMS spectral mass data, especially when looking at a wide mass range where there is clear intensity variation along the spectrum. In addition, if the data is labeled, we recommend PC-CVA instead of PCA, as it was better in determining the differences between the samples. However, there are benefits to using PCA as well as it shows a less biased view of the total variation within the data set. RF can be used to identify variables of importance in explaining sample differentiation, while its recommended to use PC-CVA first as it provides a more interpretable first view of sample differentiation in its score plot. This becomes especially beneficial when looking at large numbers of samples, for example, if screening a bacterial library for changes in many mutants. PC-CVA can then be used as the first look at cell differentiation, and then upon finding mutants of interest, look at the result of the RF to generate peak lists that can be assigned and investigate biological relevance. While PCA was susceptible to increased nonbiological variation confounding the analysis when additional data acquired over several months was included, both PC-CVA and especially RF benefited from the additional data. However, the complexity of the loadings and importance from PC-CVA and RF respectively highlight the difficulties of interpreting the results. Using both methods, one can produce peak lists of compounds that contribute to variation between the samples. However, it might only provide a direction for further targeted investigations to elucidate the biological mechanisms without prior knowledge of your sample. It is also worth noting that a seemingly big peak in a loading does not necessarily equate to a statistically significant change.

The combination and comparison of peaks lists generated from PC-CVA and RF are interesting approaches as we get both benefits of interpretability from the PC-CVA with the power of the machine learning of Random Forests.

The combination of PC-CVA and RF allowed chemical changes to be elucidated from biologically very similar samples, where no direct perturbation of membrane lipid chemistry was expected. With this approach to the analysis of GCIB-SIMS data we could quickly and easily analyze a novel data set and identify changes in cyclopropanation in an *E. coli* strain carrying the conjugative F-plasmid compared to a strain without the plasmid.

## Supporting Information Available

MATLAB commands used for the data analysis, additional data analysis plots, and peak comparison tables (PDF)
