# Supplementary material for: PCA, PC-CVA, and Random Forest of GCIB-SIMS Data for the Elucidation of Bacterial Envelope Differences in Antibiotic Resistance Research
Source: Anal Chem. 2024 Aug 20;96(35):14168–77. doi: 10.1021/acs.analchem.4c02093 (PMC11375623; doi:10.1021/acs.analchem.4c02093)
Supplement: Supplementary file 1 — ac4c02093_si_001.pdf [file ac4c02093_si_001.pdf]

Supporting information

PCA, PC-CVA and Random Forest of GCIB-SIMS data for the elucidation of bacterial envelope differences in antibiotic resistance research

Alfred Fransson[1,2], Kelly Dimovska Nilsson[1], Alex Henderson[3], Anne Farewell[1,2*], John S. Fletcher[1,2*]

1. Department of Chemistry and Molecular Biology, University of Gothenburg, 405 30 Gothenburg, Sweden
2. Centre for Antibiotic Resistance Research (CARe), University of Gothenburg, 413 45 Gothenburg, Sweden
3. Faculty of Science and Engineering, The University of Manchester, M13 9PL Manchester, U.K.

Corresponding author: John S. Fletcher, john.fletcher@chem.gu.se

Anne Farwell, anne.farewell@cmb.gu.se

Table of Contents

Supplemental Methods: S1

Supplemental Figures: S3

Supplemental Tables: S5

Supplemental Methods:

Supplemental methods 1: Matlab code.

```
x = ChiMSSpectralCollection(mass, data');
x.classmembership = ChiClassMembership('Samples', 'sample 1', n, 'sample 2', n;

%Division of ranges using keeprange;
Range1 = x.keeprange(From,to);

%Peak picking using peakdetect. Numberlimit is the max number of peaks allowed.
D2window decides the width of the area that is included when calculating the centroid
intensity;

Range1_Picked = Range1.peakdetect('numberlimit', n, 'd2window', n);

%Sum normalizes Range1 (this is used to normalized each region separately);
Norm_Range1_picked = sum_normalize(Range1_picked.data);

%This code recombines all the data after normalization and puts it into a new Chi object.
Alldata = horzcat(Norm_Range1_picked, Norm_Range2_picked,……..);
Alldata = sqrt(Alldata);
Allmass = horzcat(Range1_picked.mass, Range2_picked.mass,…….);
Allpeaks = Range1_Picked.clone;
Allpeaks.data = Alldata;
Allpeaks.mass = Allmass;

%Performs PCA analysis on Allpeaks;
PCAAllpeaks = Allpeaks.pca;

PCAAllpeaks.plotscores(1,2);
PCAAllpeaks.plotloading(1);
PCAAllpeaks.plotloading(2);


%Performs PC-CVA analysis on Allpeaks;
PC-CVA analysis:
PC-CVA = ChiPCCVA(Allpeak, (number of PC to use)):
PCCVA.plotscores(1,2);
PCCVA.plotloading(1);


%Performs the Random Forest analysis on Allpeaks;
&timed determine the number of consecutive runs that are ran and then summed up.

times = 10;
    for i = 1:times
```

```
        h = randomforest(Allpeaks,'trees', 1000);
        RFdata (i,:) = h.importances.data;
        RFpcc (i,:) = h.pcc;

    end

Mpcc = mean(RFpcc);
SumRFdata = sum(RFdata);
TimesRF = h.clone;
TimesRF.importances.data = SumRFdata;
plot(TimesRF.importances);
```

Supplemental methods 2: Workflow for the data analysis.

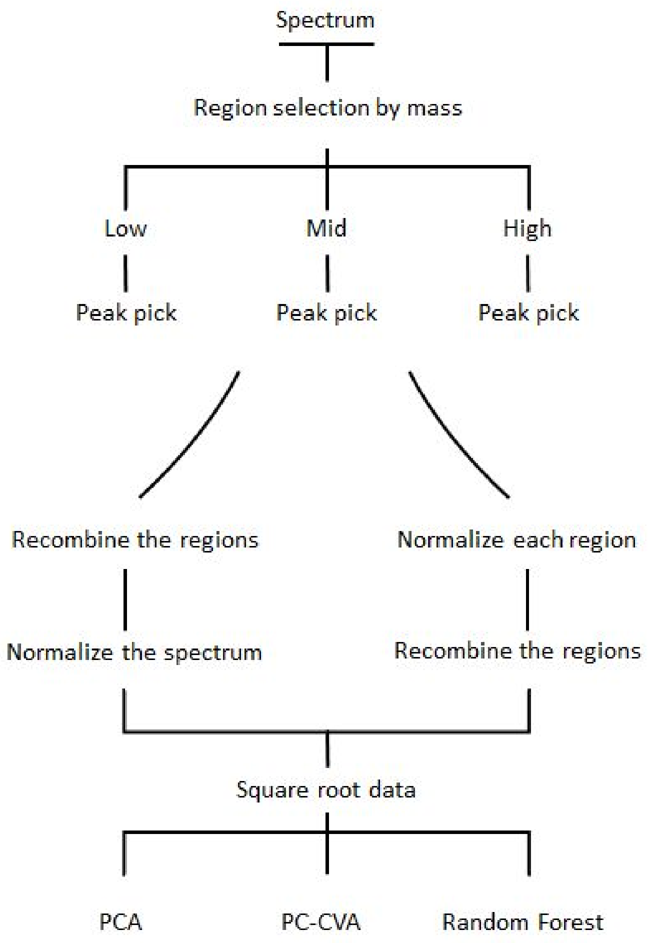

Supplemental Figures:

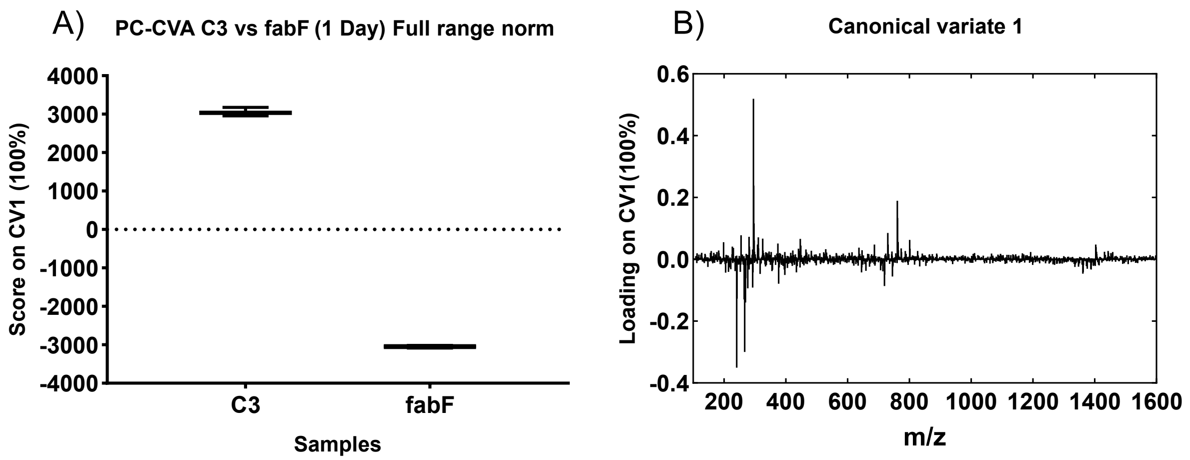

Figure S1: PC-CVA score plot (A) and loading of the canonical variate 1 (CV1) for the comparison of the wild type strain (C3) and the *fabF* deletion mutant (*fabF*). In (A) the relative score on CV1 of 8 replicates for each strain is plotted in a bar plot showing a high separation between the groups indicating a high degree of difference between then. In (B) the loading for CV1 is shown and you can see that the there is a decrease in weighing in the loading with increasing mass.

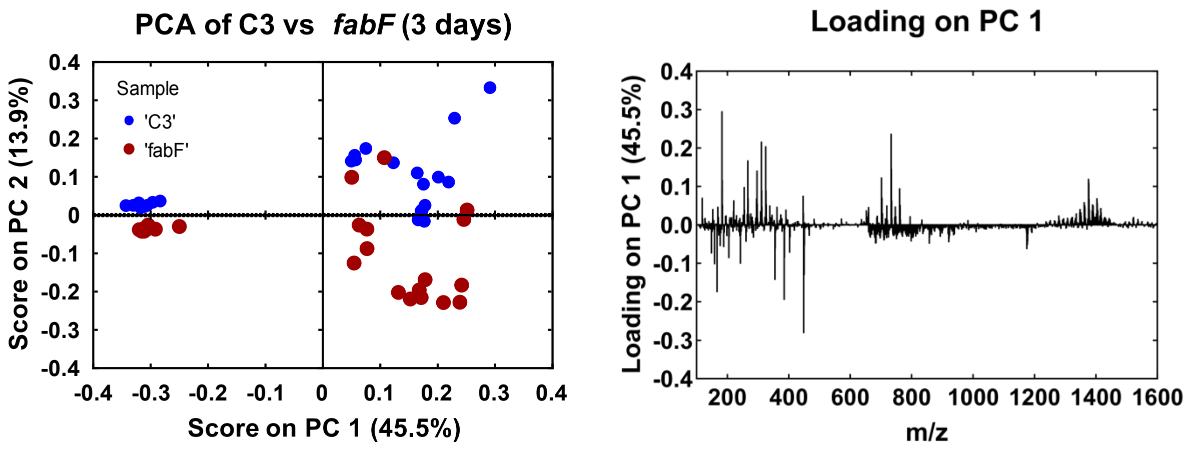

Figure S2: PCA score plot and plot of PC1 loading in the analysis of differences between the *fabF* mutant and the Wildtype (C3). The dataset used in this analysis is comprised out of data from three replicate experiment performed on three separate days, months apart. In A) PC1 separates out day 2 from day 1 & 3.

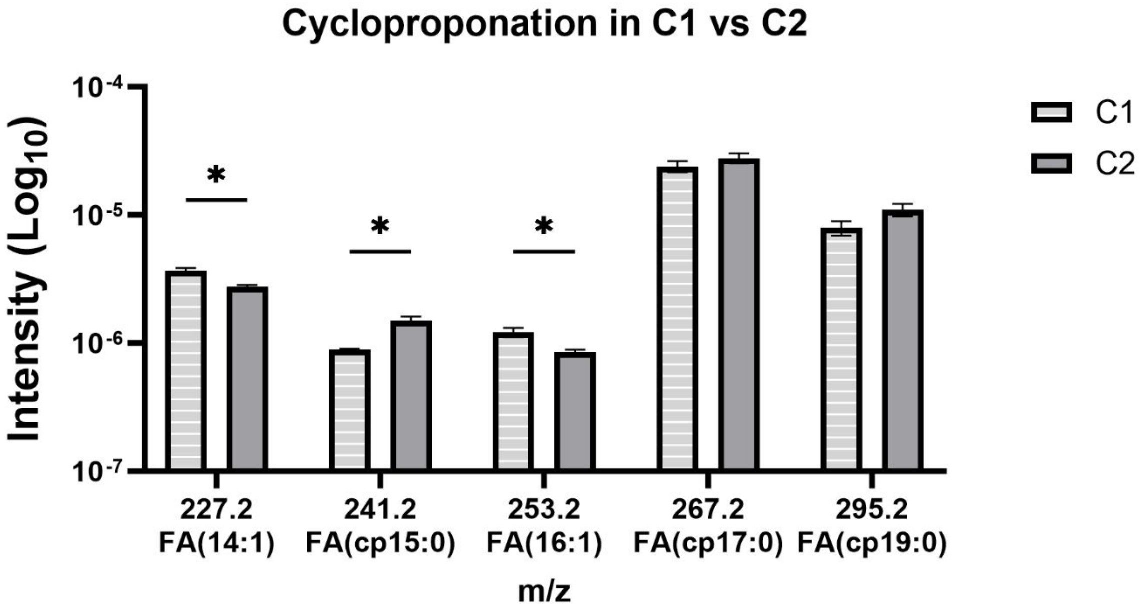

Figure S3: Bar chart of fatty acids identified in the comparison of condition C1 vs C2. The error bars are standard error of the mean (SEM) and the star (*) indicates a p-value <0.05 from a t-test.

Supplemental Tables:

Table S1: Table of the different strains and growth conditions.

Named in text	Strain name	Plasmid	Growth condition	Genotype
fabF mutant	HA42	F-plasmid (tet^{R})	-Tetracycline<br>-Kanamycin	BW25113 ΔfabF::Kan^{R}/F'Tet^{R}
C1	HA14	F-plasmid (tet^{R})	-Kanamycin	BW25113 ΔargC::Kan^{R}/F'Tet^{R}
C2	JW3930		-Kanamycin	BW25113 ΔargC::Kan^{R}
C3	HA14	F-plasmid (tet^{R})	-Tetracycline<br>-Kanamycin	BW25113 ΔargC::Kan^{R}/F'Tet^{R}

Table S2: Comparison of the top 40 hits from RF and PC-CVA of wild type (C3) and fabF mutant.

RF_Mass	RF_Values	PCCVA_Mass	PCCVA_Values
215.1088	0.04010391	241.1963	-0.130333867
§241.1963	0.100024179	255.2171	0.09632387
242.1912	0.098960332	281.2272	0.056165271
270.221	0.014149281	295.2413	0.238145037
295.0764	0.011291568	296.2501	0.10171491
295.2413	0.04432691	385.1716	0.066501291
296.2501	0.041531342	449.1357	0.099526335
377.1757	0.088409607	660.4388	-0.120837118
395.1785	0.009755518	661.4396	-0.076868782
423.145	0.011743864	665.3404	0.067208108
438.2065	0.012932813	671.3375	-0.071120529
633.3272	0.016083052	687.4612	0.075988339
645.4091	0.026043344	688.4682	-0.066432948
660.4388	0.013338238	690.4706	0.053923935
661.4396	0.022893675	707.4451	-0.075901087
687.4612	0.012114966	714.4978	-0.102252487
688.4682	0.014387188	715.4675	-0.079548027
689.462	0.01637818	717.4801	-0.076672471
707.4451	0.071890967	719.4528	-0.172786191
714.4978	0.091118155	720.4973	-0.100243649
715.4675	0.023582665	721.4566	0.071302998

716.4949	0.011652591	730.5089	0.189319236
717.4801	0.009862651	731.5037	0.123922992
719.4528	0.088126906	745.4737	-0.110471224
720.4973	0.045963774	746.4933	-0.077222881
721.4566	0.013675158	761.5024	0.399805486
722.5026	0.012092975	762.5034	0.260696234
730.5089	0.085952301	763.4903	0.11132935
731.5037	0.063982281	774.4927	0.0571715
745.4737	0.080226717	801.5121	0.063097275
746.4933	0.032661464	1348.8834	-0.067385849
761.5024	0.100963297	1361.9003	-0.123127921
762.5034	0.102912154	1362.9237	-0.110850273
763.4903	0.093972135	1375.9285	-0.106276361
795.4243	0.010980204	1376.9376	-0.11053889
801.5121	0.025425731	1387.922	-0.093406403
1361.9003	0.012486465	1388.9356	-0.092043506
1362.9237	0.02072786	1403.9613	0.063813555
1387.922	0.027854429	1404.9603	0.082708785
1388.9356	0.038266902	1405.98	0.05154903

*Green cells are found in both PC-CVA and RF

Table S3: Comparison of the top 40 hits from RF and PC-CVA of strain grown without tet (C1) and grown with tet (C3).

RF_Mass	RF_Values	PCCVA_Mass	PCCVA_Values
215.1088	0.017572426	158.9115	0.096538176
253.1935	0.010702111	166.9844	-0.160722229
254.1958	0.013428418	255.2171	-0.101186126
281.2272	0.024905308	260.845	0.108628192
282.2297	0.038115715	267.2138	-0.146870063
316.8063	0.021617703	281.2272	-0.067390804
317.8132	0.022611658	316.8063	-0.075280512
378.7931	0.031502521	378.7931	-0.064354333
379.8005	0.014895572	403.1799	0.067046369
394.7969	0.039655135	418.7467	-0.076207898
418.7467	0.113212756	447.1037	0.161336767
419.7512	0.063181453	448.1079	0.070118602
434.7304	0.063290917	660.4388	0.110726554
442.7393	0.027173974	661.4258	0.085298632
444.7388	0.027318625	664.5978	0.133126376
452.7141	0.024376524	666.6065	0.079778551
498.6899	0.011223317	671.3098	0.132952423

499.6907	0.012403698	672.6093	-0.076100787
530.6492	0.031670287	684.6117	0.067049518
536.6979	0.035083791	687.4612	0.149117468
538.6764	0.051201973	696.5656	-0.077337102
539.6794	0.026614533	702.4753	-0.125352085
540.6834	0.029551326	703.4792	-0.077313693
552.6906	0.01921249	718.5376	-0.067007065
554.6606	0.029599897	720.5259	-0.072423828
560.6802	0.024989423	733.4666	-0.103181471
564.6649	0.010679159	734.4634	-0.095749693
570.6364	0.061987206	795.4243	0.069673895
571.6688	0.049771132	814.5029	-0.071062519
576.665	0.059951639	854.4761	-0.0658103
592.6538	0.0289935	878.4612	-0.074777443
600.6387	0.032097763	921.2987	0.082512002
601.6323	0.024902916	983.5328	-0.081298944
616.6099	0.050299837	984.5031	-0.063910653
632.5893	0.055274427	1375.9285	0.12124681
641.6212	0.014580415	1376.9376	0.102869785
650.5946	0.015773938	1377.9273	0.06874587
664.5978	0.040384112	1403.9613	0.113390426
685.5469	0.010766568	1404.9603	0.109246534
983.5328	0.033721465	1405.98	0.067808772

*Green cells are found in both PC-CVA and RF

Table S4: Comparison of the top 40 hits from RF and PC-CVA of strain with the F-plasmid (C1) and without plasmid (C2).

RF_Mass	RF_Values	PCCVA_Mass	PCCVA_Values
157.9047	0.006536003	158.7704	0.081251
158.9115	0.007815433	158.9115	0.272523
227.1857	0.013371271	176.9202	0.105368
228.1834	0.010276169	227.1857	0.087148
241.1963	0.018920323	241.1963	-0.09186
242.1912	0.00851756	260.845	0.063076
253.1935	0.008850794	267.2138	-0.07045
343.1303	0.012232115	295.2413	-0.12604
344.1385	0.010358854	339.1707	-0.0597
377.1653	0.009716605	447.1037	0.070208
395.1678	0.005407055	671.3098	0.138866
439.1559	0.006249264	673.3978	-0.0711
443.1882	0.006134581	681.3088	0.065545

482.7191	0.005220273	685.5469	-0.06065
498.1779	0.008429316	690.4567	0.088278
502.7231	0.010040268	691.4238	0.063419
504.722	0.005466004	707.4734	-0.08061
511.1881	0.00654145	721.4566	0.081909
518.7029	0.006299998	726.5649	-0.0661
522.6943	0.00960596	730.4944	-0.05831
523.6945	0.005689983	733.4666	0.246055
534.6984	0.006953842	734.4634	0.146251
559.2164	0.004991816	735.4755	0.091122
568.7018	0.005374244	787.5029	-0.06284
581.683	0.009347332	794.4771	-0.05885
582.6867	0.010329179	798.4954	0.11559
584.6579	0.007174413	807.4867	-0.06305
586.6583	0.006155208	891.4059	-0.0608
602.6531	0.011998369	921.2987	-0.06192
603.6615	0.009507262	987.467	-0.0657
606.6526	0.005753736	1225.7936	0.070091
622.6497	0.011107159	1361.9003	-0.06601
624.634	0.009531651	1362.9237	-0.0689
636.6195	0.005263441	1364.9324	0.063078
643.6354	0.017841348	1375.9285	0.069606
644.6235	0.005337443	1376.9376	0.063634
999.1142	0.005278608	1389.9493	-0.08846
1226.8021	0.02245849	1390.9436	-0.0858
1343.288	0.005139544	1417.983	-0.06398
1527.0977	0.008534585	1527.0977	0.074609

*Green cells are found in both PC-CVA and RF
